# Energetic and Monetary Analysis of Efficiency in Family-Owned Dairy Goat Production Systems in Andalusia (Southern Spain)

**DOI:** 10.3390/ani14010104

**Published:** 2023-12-27

**Authors:** Yolanda Mena, Eduardo Morales-Jerrett, Marta Soler-Montiel, David Pérez-Neira, Juan Manuel Mancilla-Leytón

**Affiliations:** 1 Departamento de Agronomía, Escuela Técnica Superior de Ingeniería Agronómica, Universidad de Sevilla, 41013 Sevilla, Spain; edumorjer@alum.us.es; 2 Departamento de Economía Aplicada, Universidad de Sevilla, 41012 Sevilla, Spain; msoler@us.es; 3 Departamento of Economía y Estadística, Universidad de León, 24071 León, Spain; dpern@unileon.es; 4 Departamento de Biología Vegetal y Ecología, Facultad de Biología, Universidad de Sevilla, 41012 Sevilla, Spain; jmancilla@us.es

**Keywords:** caprine, eco-efficiency, energy metabolism, feed management, LCA

## Abstract

**Simple Summary:**

Dairy goat farming in Andalusia is diverse and predominantly family-based and presents particularities in terms of its economic strategies that require a specific approach. For this reason, in the context of climatic and energy crises that have a decisive influence on the activity, it has been considered relevant to deepen the knowledge of the economic and energy profiles of the different goat production systems, filling an information gap that existed. For this purpose, twenty-one farms, representatives of the dairy goat system diversity in Andalusia, were monitored for one year, obtaining technical-economic information that allowed the calculation of different energy and economic indicators. The results of this work demonstrate that, from an energetic point of view, goat farms that base their feeding strategies on grazing and make optimal use of natural resources are more efficient than models based on permanent stabling. This greater environmental sustainability derived from extensification is penalized by the markets, which give priority to intensive models with greater milk production and less food autonomy. Nevertheless, this study demonstrates that extensification strategies are capable of remunerating family labor and can, therefore, be economically viable, provided that support from the Common Agricultural Policy is included in the analyses.

**Abstract:**

The family-owned dairy goat sector in Andalusia presents great diversity. Taking into account the particularities of their economic strategies, which are focused on generating net value added and a stable long-term remuneration for family labor, this work aims to expand the scarce existing knowledge on the energetic and economic profiles of the different caprine management systems in a context of climate and energy crisis. For this purpose, twenty-one farms, representatives of the four typologies of the Andalusian dairy goat system, were monitored for one year: pastoral systems, grazing systems with high feed supply, indoor systems with associated crops, and indoor systems without associated crops. Technical-economic information was obtained that allowed the calculation of energy and economic indicators. In terms of socialized output, the differences found were due to the energy derived from milk sales, which was clearly lower in pastoral systems. The higher proportion of energy output obtained from manure with respect to edible products (milk and meat) highlights the importance of the former in energetic terms. High values for external inputs are found in the intensive group (111.22 GJ LSU^−1^), while the lowest results correspond to the pastoral group (36.96 GJ LSU^−1^). The main external input is the energy proceeding from purchased feed, which accounts for over 79% of the total external energy input in all four groups. The highest energy efficiency corresponds to the pastoral group, which is also the most efficient one in the use of non-renewable energy to produce milk and meat. Additionally, the level of eco-efficiency is higher in pastoral systems. Common Agricultural Policy funds contribute to increasing the remuneration of family work in pastoral systems, assimilating it to the rest of the systems. Therefore, intensification does not imply an absolute monetary advantage in all cases, while extensification can be remunerative for family-owned dairy farming.

## 1. Introduction

Dairy goat rearing has great socio-economic and environmental importance in Mediterranean countries because it provides food of organoleptic, functional, and nutritive quality [1], generates employment and economic resources in marginal areas, reverses depopulation in rural areas [2,3], and promotes a circular economy. In addition, when there is grazing, goat rearing contributes to seed dispersal, biodiversity enhancement, natural fertilization, and forest fire prevention, among other benefits [4]. These advantages are closely related to the different management systems implemented, particularly in family farms. Being acquainted with and understanding this diversity is, therefore, of increasing scientific relevance [5].

In the last few decades, goat production systems in Europe have experienced important technological advances in management and genetic improvement, which have led to an increase in productivity per animal and farm, the optimization of labor and other resources, and the generation of health-safe products [6]. These improvements have come along with a significant process of production intensification [7,8], aimed to increase the performance per goat and year, particularly in milk production, because in most cases, milk sales amount to 80% of the farms’ income [9]. This process has progressively broken the link between this sector and the territory where it is based, with important social and environmental consequences.

Despite this intensification and in contrast with what has happened in other sectors, such as the dairy cow sector and, to a lesser extent, the sheep sector, the Spanish dairy goat sector is very diverse in its production systems. This diversity is especially evident in Andalusia, the Spanish southern region, which has a population of 0.99 M goats and produces 51% of the country’s goat milk and 13.26% of the European output [10]. Practically, the whole production comes from local breeds (Murciano-Granadina, Malagueña, Florida, and Payoya) that are perfectly adapted to their environment and managed under different production systems. The systems range from those where goats remain indoors throughout the year, although they have access to outdoor exercise yards [11,12], to those in which the goats graze all year long and most of the day [5,13]. The farms are generally managed by a family labor force and provide economic sustenance to families who live mostly in disadvantaged areas with very few economic and production alternatives [14].

In Europe, the dairy goat sector has been experiencing economic viability problems for decades, although the situation varies greatly depending on the country [6,8,15,16]. This economic crisis reduces the ability to improve sustainability, especially in family-owned farms (goat family farms) [17,18], and creates dependence on public support [19]. Given the difficulty of influencing selling and the main input prices, the focus is on cost reduction [20]. Feed and labor costs appear as the main costs in all studies, together with the cost of indebtedness linked to fixed-capital investments in modernization and intensification processes [21]. Some research indicates that reducing feed costs linked to grazing can increase gross margin [13,22]. Other works indicate that, without proper management, the reduction in feed costs either does not exist or can be offset by increased labor costs [18]. There is also an open debate on the most appropriate indicators to assess the sustainability and economic viability of family farming enterprises [23,24]. In any case, it should be noted that studies on the economic viability of dairy goat farms (farm-level economic viability) in Europe are very scarce, especially those that differentiate between production systems according to their degree of intensification, despite repeated references to the high heterogeneity existing in the activity [6,8,16]. In the case of family farms, it is necessary to take into consideration the particularities of their economic strategies, focused on generating net value added and a long-term steady remuneration for the family’s own work, as their way of life through various management systems or styles of farming [24,25].

From the point of view of sustainability, debates on the economic viability of goat rearing are complicated with analyses that allow a better understanding of the dependency on and the efficiency of natural resources. In the current climate change and peak oil context, this is particularly important in relation to the use of energy [26,27,28]. Thus, the energy transition and climate change adaptation processes are, among other issues, central and cross-sectional elements of the New Green Deal [29], which urges the scaling up of food-provision systems based on the use of renewable energy, low emission intensity, and high energy efficiency in the production of biomass [30]. In this new context, efficient agricultural or stockbreeding systems will not only be the most productive ones but also the ones capable of optimizing the input of useful energy and of channeling it, to a larger extent, to the production of usable goods, mostly food and manure, and of derived ecosystem services [31].

The Spanish Plan for Climate Change Adaptation (2021–2030) and the more local Andalusian Plan for Climate Action (2021–2030) are defined on the same terms, indicating the need to increase knowledge on the assessment of global warming risks and impacts on the main crops and livestock species and, on that basis, to improve sustainability and climate change adaptation in rural areas by promoting short marketing channels, bioeconomy, circular economy, and proximity agriculture, among other strategies meant to mitigate climate impact and improve resilience [32,33]. This is why, for sound individual and sectorial decision-making, it is necessary to increase knowledge of the different production systems, not only from an economic perspective but from a biophysical one, based on the use of specific indicators. These indicators should be objectively verifiable, replicable, sensitive to changes in the system, and capable of analyzing their relationship with other indicators. In addition, the information collection process to generate these indicators must be simple and non-costly for the farmers [34].

In the case of dairy goat rearing, the literature has mainly focused on calculating and analyzing greenhouse gas emissions (GHG) through the sector’s carbon footprint [17,35,36,37,38]. From the point of view of sustainability, some works have shown how organic production can reduce the GHG emissions of the industry [39], especially if carbon sink is taken into consideration [40,41], while other studies have researched the potential of reducing the emissions associated with grazing [42]. There are fewer studies that analyze the use of energy in this context [43]. Some of them quantify energy consumption in goat milk production as part of a wider assessment linked to the life cycle analysis (LCA) methodology [44]. For instance, Zucali et al. [45] and other authors have shown how enteric emissions and manure storage are the main hotspots in Italian farms in terms of carbon footprint [35,40]. However, feed and, particularly, purchased fodder are the main hotspots in other impact categories, such as freshwater eutrophication, soil use, and depletion of fossil and renewable mineral resources (/ib). In the same vein, Soares-Cabral et al. [46] analyze how the partial replacement of soya with grass or hay may be an important driver of reduction in energy consumption.

From a methodological point of view, what these works have in common is that they analyze the energy dimension in terms of the farms’ “expenses” or “costs”, but they do not include any efficiency indicators such as, for example, the energy return on energy invested (EROI) [47]. Recently, some authors have criticized energy analyses as insufficient because they reproduce the economistic cost–benefit logic and have proposed alternative approaches to measure “hidden” energy flows and the energy-related opportunity costs, such as for instance, those linked to the use of manure or to grazing [48,49]. These internal energy flows, which are usually neither accounted for nor analyzed for not being market-oriented, contribute to the maintenance of the structure and functions of agro–silvo–pastoral systems and are, therefore, fundamental in the flow of ecosystem services [49,50]. As far as the authors know, there is only one previous work that has applied this approach to the study of the dairy goat sector at the farm level, focusing on only three farms [51]. But, it does not consider the monetary dimension of production, and it is therefore difficult to carry out comparative analyses of the results concerning this activity and of the various production models. This work aims to contribute to filling this gap by presenting, in a novel way, the energy and economic profiles of the different management systems used in the dairy goat-rearing sector.

The main objective of this work is to analyze, from an energy and monetary perspective, the efficiency of various dairy goat production systems using local breeds, classified according to their degree of intensification. The purpose is to contribute to the generation of knowledge on the economic, social, and environmental dimensions of this activity. For the achievement of this general objective, the authors suggest filling the existing information gap through (i) generating energy indicators using an agroecological approach that takes into account the farms’ internal energy flows; (ii) generating monetary indicators to assess the economic dimension of each production model; and (iii) analyzing the behavior of the different dairy goat production systems in terms of eco-efficiency. This knowledge is necessary to develop dairy goat production systems that are capable of responding appropriately to the current needs of Western societies, not only in regards to the provision of enough quality food but also as an element that can improve the natural environment in which the farms carry out their activity and provide a livelihood for people living in rural areas.

## 2. Materials and Methods

### 2.1. Sample Selection and Data Collection

Twenty-one representative dairy goat farms using local breeds in Andalusia (southern Spain) were selected and monitored for one year (2018) in order to collect information that allowed the generation of technical and economic indicators following a methodology developed by the authors in previous studies [13,52]. Attending to the criteria established by Morales-Jerrett et al. [5], the farms were classified into four groups: (i) IS: indoor systems without associated crops (n = 8); (ii) ISC: indoor systems with associated crops (n = 3); (iii) GS: grazing systems with high feed supply (n = 5); and (iv) PS: pastoral systems (n = 5). Table 1 shows the main characteristics of each group of study. The main general data gathered from the four dairy goat production systems can be found in Table S1 and Figure S1 of the Supplementary Materials.

### 2.2. Energy Assessment

#### 2.2.1. Boundaries, Functional Units, and Analysis Perspective

For the energy analysis carried out in this study, a “cradle-to-farm gate” approach was implemented. The main functional unit used was the livestock standard unit (LSU; one adult animal = 0.15 LSU, the rest of animals = 0.11 LSU), with the liter of milk produced used as a complement. The agroecological approach proposed by Pérez-Neira et al. [51] for the energy analysis of caprine activity is applied here because it contemplates stockbreeding as part of a complex agroecosystem exchanging energy flows with other natural and social systems [50,53]. This approach brings to light and analyses internal energy flows that are not usually taken into consideration despite their contribution to maintaining the structure and functions of the ecosystem and, therefore, sustaining the flow of ecosystem services [49,54].

**Figure 1:**
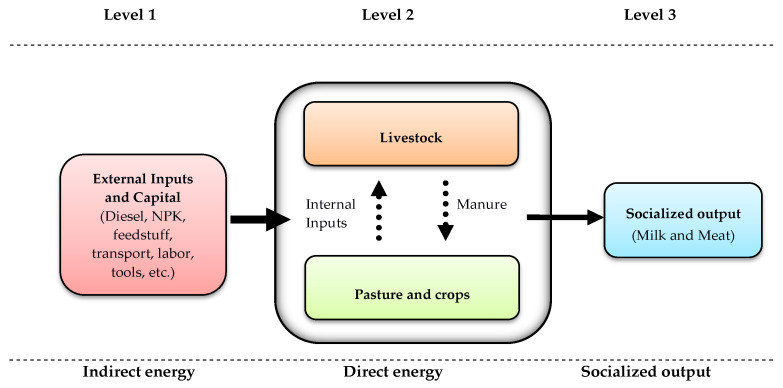
System boundaries of the energy analysis.

The analysis was structured around three levels: (1) it incorporated the indirect energy cost of producing and transporting the inputs and capital used during the livestock production process; (2) it took into consideration the energy directly consumed inside the farm; and (3) it included the energy and capital generated by the milk and meat produced, known as socialized output (Figure 1). Given that two of the four types of farms studied here have implemented models in which the goats are permanently indoors, and considering the heterogeneity of the territories in which goats graze in the other two models (Figure S1 of the Supplementary Materials), such variables as unharvested biomass (the biomass that returns to the system by abandonment, without human intervention) and the accumulated biomass of woody species are not included in the analysis, as suggested by Ramos-García et al. [49].

#### 2.2.2. Energy Indicators

Ten indicators were selected for the energy analysis. Total energy output (TEO) accounts for livestock-related energy output, whether socialized (milk or meat), re-employed (manure), or part of the livestock structure (increase/decrease in herd weight) (Equation (1)). The cumulative energy demand (CED) measures the use of energy in livestock farms, incorporated in the form of external inputs (concentrates, fertilizers, diesel, machinery, etc.) (EI) or internal inputs (own crops for livestock, grazing, etc.) (II) (Equation (2)). The coefficients used to calculate the energy output (EO) were taken from Moreiras et al. [55] and Pérez-Neira et al. [47], while the CED was calculated using the CML-IA methodology (3.07 version) and the Ecoinvent 3.5 and Agribalyse 3.0 databases with SimaPro software (9.3.0.3).

TEO = ∑ SO × α_(o)_ + EOr × α_(e)_ + I/D L × α_(id)_ (1)

CED = ∑ EI + II = ∑ I_(j)_ × ß_(j)_ (2)

TEO = total energy output (MJ LSU^−1^); SO = socialized output (unit LSU^−1^), which includes milk (l LSU^−1^) and meat (kg LSU^−1^); α(o) = energy coefficient of socialized output (MJ L^−1^ or kg^−1^); EOr = energy output reuse (manure or milk) (kg LSU^−1^); α(e) = energy coefficient of manure or milk (MJ kg^−1^); I/D L = energy increase/decrease in the number of livestock units (kg LSU^−1^); α(id) = energy coefficient of increase/decrease in livestock (MJ kg^−1^); CED = cumulative energy demand (MJ LSU^−1^); EI = external inputs: concentrates, fodder, electricity, diesel, labor, and other expenses, etc. (MJ LSU^−1^); II = internal inputs: own crops consumed during grazing or indoors, and energy contribution of natural pastures (MJ LSU^−1^); I(j) = Input “j”, where j = feed purchased, fodder, electricity, petrol/diesel, lubricants, phytosanitary material, plastics, tools, fertilizers, seeds, machinery, labor, etc. (unit LSU^−1^); and ß(j) = energy coefficient of I(j) (MJ unit^−1^).

The final energy return on investment (final EROI) assesses the energy efficiency of livestock systems (Equation (3)), especially in relation to the use of non-renewable energy (NR final EROI) (Equation (4)). The NR EROI_food_ (Equation (5)) focuses on the energy efficiency of producing human food. The external feed dependence (EFD) (Equation (6)) measures the degree of intensification/extensification as the level of dependence/autonomy of a farm in relation to purchased livestock feed. The avoided energy cost of manure (AECM) measures the savings derived from replacing synthetic fertilizers with manure (Equation (7)), while the avoided energy cost of natural pasture consumed during grazing (AECP) does so in relation to the use of natural pastures (Equation (8)). This index is generated by adding to the gross energy provided by natural pasture (ECNP) the energy cost of production required to obtain the same amount of energy (EC of ECNP) from cultivated crops. Complementarily, by applying the concept of opportunity cost, the food/feed EROI (Equation (9)) measures the energy efficiency of transforming edible human food into edible energy in the form of meat and milk.

Another relevant concept to be integrated into energy analyses of livestock husbandry is that of “avoided cost”. It allows for identifying the benefits derived from choosing one alternative instead of another. This concept was initially proposed by Environmental Economics as a monetary indicator [56], and it has been recently reinterpreted from a biophysical perspective by Ecological Economics. The avoided land cost of natural pasture consumed during grazing (ALCP) has been calculated based on the agricultural area required to produce the feed (grains, feed concentrates, and cultivated fodder) that would be necessary to find a substitute, in terms of energy, for the contribution of natural pasture consumed by grazing (Equation (10)).

Final EROI = TEO × CED^−1^ (3)

NR Final EROI = TEO × NR CED^−1^ (4)

NR EROI_food_ = (SO × α_(o)_)/NR (5)

EFD = GEEF × GERL^−1^ × 100 (6)

AECM = RM × N_m_ × ß_(N)_ (7)

AECP = ECNP + EC of ECNP(8)

Food/feed EROI = SO × GEfeed_g/c_^−1^ (9)

ALCP = ECNP × EYsc^−1^ (10)

Final EROI = final energy return on investment; TEO = total energy output (MJ LSU^−1^); CED = cumulative energy demand (MJ LSU^−1^); NR final EROI = non-renewable final EROI; NR CED = non-renewable CED (MJ LSU^−1^), to calculate this indicator, the use of renewable energy was subtracted from the CED; NR EROI_food_ = NR EROI related to food production; SO = socialized output (unit LSU^−1^), which includes milk (L LSU^−1^) and meat (kg LSU^−1^); α(o) = energy coefficient of socialized output (MJ L^−1^ or kg^−1^); EFD = external feed dependence (%); GEEF = gross energy of external feed (MJ LSU^−1^); GERL = gross energy requirements of the livestock^−1^ (MJ LSU^−1^); AECM = avoided energy cost of manure (MJ LSU^−1^); RM = reused manure (kg LSU^−1^); Nm = nitrogen contained in manure (kg N kg^−1^); ß(N) = energy coefficient relative to the energy cost of producing nitrogen (MJ kg N^−1^); AECP = avoided energy cost of natural pasture consumed during grazing (MJ LSU or L^−1^); ECNP = energy contribution of natural pasture consumed during grazing (MJ LSU or L^−1^); EC of ECNP = energy cost of production to generate ECNP if it were replaced by crops (MJ LSU or L^−1^); Food/feed EROI = food/feed energy return on investment; SO = socialized output (MJ LSU^−1^); GEfeedg/c = gross energy of grains/feed concentrates with an opportunity cost with regard to human food (MJ LSU^−1^); ALCP = avoided land cost of natural pasture consumed during grazing (ha); and EYsc = energy yield of the substituted crops (MJ ha^−1^).

### 2.3. Economic Indicators

There is a theoretical-methodological debate on the most appropriate indicators to assess and perform comparative analyses of the economic viability and sustainability of farms. Spicka et al. [23] suggest the value added and the value added per unit of work because these indicators represent the remuneration of the factors of production (land, labor, and capital) irrespective of their ownership, as unpaid family labor is not deducted. In the case of owned-family farms, Rossi [57] recommends analyzing the family farm income, defined as the financial reward to all members of the family who work on the farm for their labor, management, and investment, i.e., the remuneration of family labor, land, and capital factors. This question is especially relevant in the analysis of family farms based on the unpaid labor of family members, as farming is a way of living and not only a self-employment strategy [24]. Thus, the most accurate indicators to assess the economic viability of family farms are the net value added and the family farm income (total and per worker) calculated after the deduction of the cost of paid labor [58], which represents the return to the farmer family for the use of their own production factors [57]. The family farm income is equivalent to the net margin when the cost of family labor and other opportunity costs are not subtracted from its calculation.

The revenues (Equations (11) and (12)) are milk, goat kids, animals for rearing, and other sources of income, such as manure and aids. The main costs (Equations (13) and (14)) included are feed, health, energy, hired labor, amortizations, and other minor costs. Based on this information, six economic indicators were obtained. The gross value added (GVA) (Equation (15)) and the net value added (NVA) (Equations (16) and (17)) result in resting to the market incomes the intermediate consumption in the first case and also the amortizations in the second. The goat family farm income (GFFI) (Equation (18)) represents the remuneration of the family labor. When aids are taken into account, the goat family farm income cap aid (GFFI_CAP aid_) is calculated (Equations (19) and (20)). Furthermore, using the CED, two eco-efficiency indicators were generated: one in relation to total market revenues, the EI1 (Equation (21)), and the other in relation to the goat family farm income with aid (GFFI_CAP aid_), the EI2 (Equation (22)).

SI = SM +SGT+ SOG + SAR+ SM_manure_ (11)

AI = DP_CAP_ + RDP_CAP_ (12)

IC = FC + HC+ EC + MC(13)

HLC = W × HWD(14)

GVA = SI − IC(15)

NVA = GVA − AM(16)

NVA_per worker_ = NVA × AWU^−1^ (17)

GFFI = SI − (IC + HCL + AM)(18)

GFFI_CAP aid_ = TI − (IC + HCL + AM) (19)

GFFI_CAP aid per worker_= GFFI_CAP aid_ × AWU^−1^ (20)

EI1 = SI × NR CED^−1^ (21)

EI2 = GFFI_CAP aid_ × NR CED^−1^ (22)

SI = total market incomes (EUR goat^−1^) that include the sales of milk (SM), goat kids (SGK), old goats (SOG), animals for rearing (SAR), and manure (SM_manure_); AI =aAid income (EUR goat^−1^) result of adding Direct Payment (DP_CAP_) and Rural Development Payment (RDP_CAP_) of Common Agrarian Policy; IC = intermediate consumption (EUR goat^−1^) that includes cost of feed (FC), health (HC), energy (EC), and other minor costs (MC); HLC = hired labor cost (EUR goat^−1^) that is the result of multiply wages (W) by hired working day (HWD); GVA = gross value added (EUR goat^−1^); NVA = net value added (EUR goat^−1^ or EUR AWU^−1^); GFFI= goat family farm income (EUR goat^−1^); AM = amortizations (EUR goat^−1^); GFFICAP aid = goat family farm income with aid (EUR goat^−1^); GFFIcap aid per worker = estimates the above indicator expressed per worker (EUR AWU^−1^); EI1 and EI2 = eco-efficiency indicators (EUR GJ^−1^); NR CED = non-renewable CED (MJ LSU^−1^), to calculate this indicator the use of renewable energy was subtracted from the CED.

### 2.4. Statistical Analysis

To test for possible significant differences among the results of the four dairy goat production systems, an analysis of variance (ANOVA) was carried out. This was preceded by a test of normality and homoscedasticity. Variables have been log10-transformed for normalization of frequency distribution where necessary. A Tukey test was performed to evaluate significant differences between them (*p* ≤ 0.05) in two-to-two comparisons. For this purpose, IBM SPSS v 25.0 was used.

## 3. Results

### 3.1. Energy Assessment

#### 3.1.1. Energy Outputs and Inputs of the Caprine Activity

**Figure 2:**
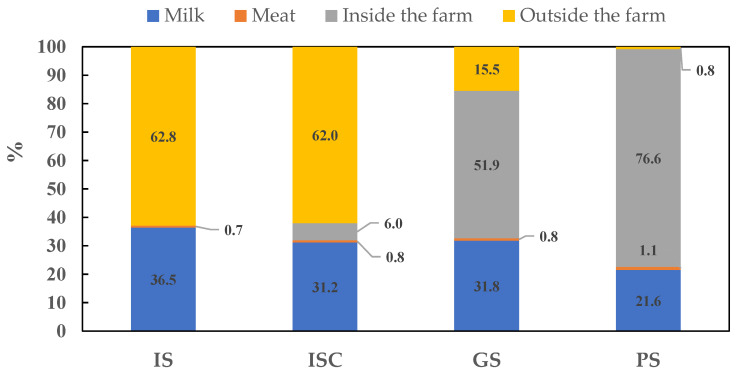
Proportion of the components of the total energy output (TEO) in the four dairy goat production systems identified in Andalusia, excluding accumulated biomass and reused milk inside the farm. IS: indoor systems without associated crops; ISC: indoor systems with associated crops; GS: grazing systems with high feed supply; and PS: pastoral systems. Socialized output: milk and meat. Reused biomass: manure used inside and outside the farm (inside and outside the farm in the figure).

The main energy outputs of the monitored farms are shown in Table 2. Significant differences in terms of total energy output were not found in two of the elements analyzed: the socialized output (SO) and the energy output reused (EOr). The accumulated biomass and the increase/decrease in the number of animals during one year had no influence on the final results because of their low value. In terms of socialized output (SO), the levels of energy obtained from meat were similar in all groups, and the differences found were due to the energy derived from milk sales (*p* = 0.041), which was clearly lower in pastoral systems (5.34 GJ LSU^−1^, Table 2). When the energy output reused was analyzed, significant differences were found between the indoor system without associated crops (IS) and the rest of the groups (*p* = 0.030) due to differences in the management of manure. In the systems that included grazing, i.e., grazing systems (GS) and pastoral systems (PS), 74.36% and 95.35%, respectively, of the energy output was related to the use of manure inside the farms, unlike what happened in indoor farms (IS and ISC), where manure was carried outside the farm and represented up to 100% and 91.24%, respectively, of the total biomass energy reused (Table 2). Figure 2 represents the proportion of the different energy outputs described in Table 2. Differences between inside and outside use of manure are shown, as is the importance of energy proceeding from milk. This figure is useful to highlight the proportion of energy output obtained from manure (biomass reused outside or inside the farm) and from milk and meat sales. This proportion varies from 63:37 in the IS group to 77:23 in pastoral farms. In the ISC and GS groups, the proportion is, respectively, 68:32 and 67:33.

**Figure 3:**
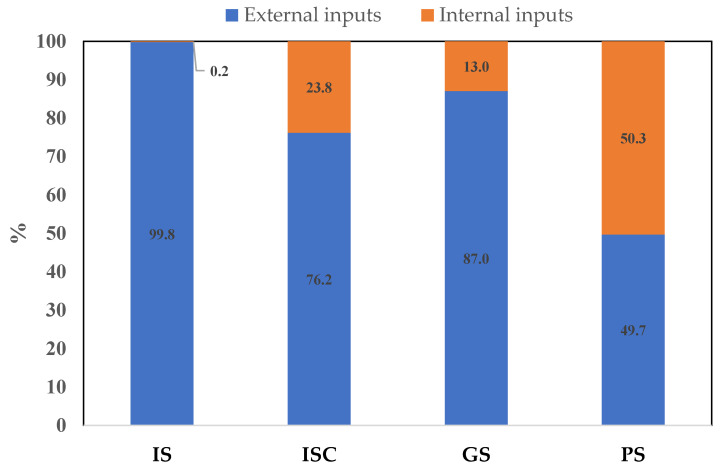
Proportion of the components of the cumulative energy demand (CED) in the four dairy goat production systems identified in Andalusia. IS: indoor systems without associated crops; ISC: indoor systems with associated crops; GS: grazing systems with high feed supply; and PS: pastoral systems.

**Figure 4:**
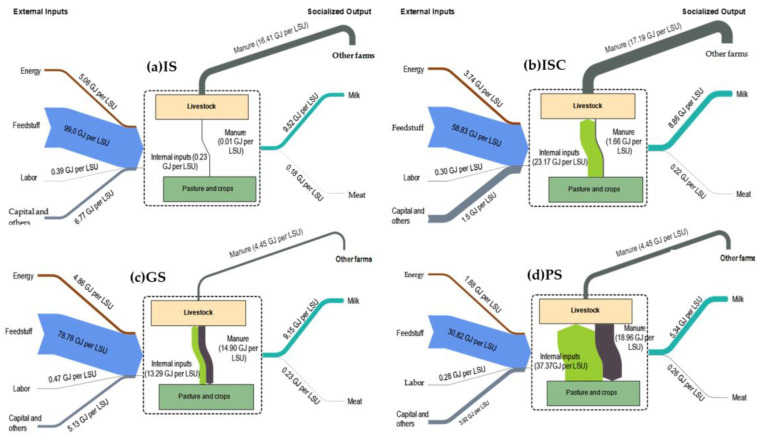
Energy metabolism of the four dairy goat production systems identified in Andalusia. IS: indoor systems without associated crops (**a**); ISC: indoor systems with associated crops (**b**); GS: grazing systems with high feed supply (**c**); and PS: pastoral systems (**d**).

Unlike the outputs, the energy inputs of the different farming systems present significant differences, as shown by the two components of the CED values: external and internal inputs (Table 3). High values for external inputs were found in the IS group (111.22 GJ LSU^−1^), while the lower results corresponded to the PS group (36.96 GJ LSU^−1^). The main external input is the energy proceeding from purchased feed, which accounts for more than 79% of the total external energy input in all four groups and for which significant differences were found between systems. No differences were found in the categories of electricity, diesel/gas, labor, and other operational expenses (Table 3). The agroecological approach incorporates into the energy analysis the farm crops used to feed the goats and the natural surfaces used for grazing. Obviously, IS values are close to 0.00, and there are significant differences between groups. Energy from cultivated forages is important in the ISC group (23.17 GJ LSU^−1^), while the GS and the PS groups present high values of energy linked to grazing surfaces, particularly when they are natural (8.39 and 29.55 GJ LSU^−1^, respectively) (Table 3). As a result, the proportion of internal and external energy inputs varies according to the system reviewed (see Figure 3). The different goat production systems analyzed have diverse energy metabolisms—(Figure 4).

#### 3.1.2. Energy Efficiency Indicators and Energy Costs Avoided in the Dairy Goat Farms

In terms of energy efficiency, significant differences were found between systems when applying the final EROI and NR final EROI indicators. The highest energy efficiency corresponds to the PS group (0.34 final EROI and 2.43 NR final EROI) (Table 4). Likewise, pastoral systems are the most efficient ones in the use of non-renewable energy to produce milk and meat (NR EROI_food_). On the other hand, it is evident that the degree of dependence on external animal feed is very high in the IS group, where concentrates and forage were all purchased (99.66%), while pastoral systems have the lowest dependence (36.57%). It is worth noticing that the GS group, with an important grazing component, shows a high degree of dependence (82.55%). Finally, the ISC group exhibits a dependence rate of 66.82% due to the use of cultivated forage to feed the animals (Table 4).

The reuse of manure and the consumption of natural resources through grazing are important sources of energy saving (Table 4). There were significant differences in the costs avoided by the reuse of manure (AECM) between grazing (GS, PS) and indoor systems (IS, ISC). This is obviously also true of the use of natural pastures (AECP), where the avoided cost in the two indoor systems is 0.00. Finally, to determine energy efficiency in relation to human food competition, two indicators were calculated: the food/feed energy return on investment (food/feed EROI), where no differences are found between groups, and the avoided land cost of natural pasture consumed during grazing (ALCP), where representative values are described only for the two systems with grazing (2.96 ha LSU^−1^ or 1.64 ha 1000 L^−1^) (Table 4).

### 3.2. Economic Analysis

The results of the economic analysis for each type of farm are shown in Table 5. With regard to income, significant differences are found both for income proceeding from the sale of milk and meat (socialized output income) and for income obtained from financial support (aid income). In the first case, pastoral systems have a lower level of income than the rest of the groups, while in the second, intensive farms with no territorial basis are the ones receiving less community aid funds. On the other hand, significant differences are found when intermediate consumption (IC) is compared between systems; pastoral systems (PS) have a significantly lower IC (Table 5).

**Figure 5:**
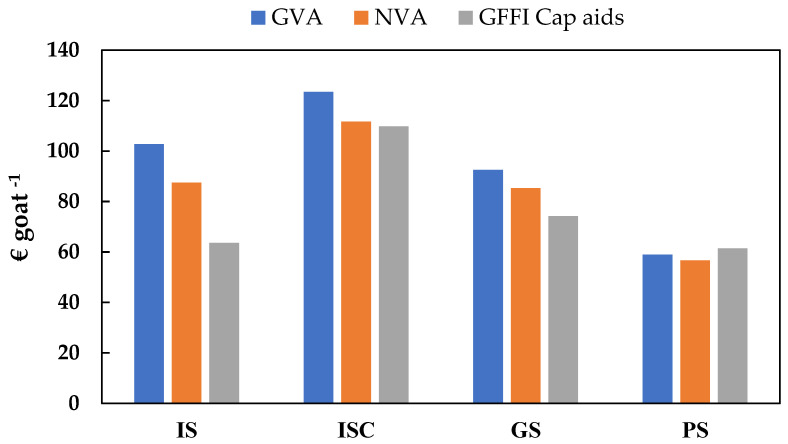
Gross value added (GVA), net value added (NVA), and goat family farm income with aid (GFFI_CAP aid_) for each production system. IS: indoor systems without associated crops; ISC: indoor systems with associated crops; GS: grazing systems with high feed supply; and PS: pastoral systems.

Although, statistically, there are no significant differences between the economic efficiency indicators analyzed, the results show a tendency toward a greater capacity to remunerate the workers in intensive farms (IS and ISC), as well as a higher level of investment to increase production (Table 5 and Figure 5). Likewise, the capacity of the market to remunerate family labor tends to be smaller in pastoral systems when aid income is not taken into consideration (29.08 EUR goat^−1^). It is evident that, in the caprine sector, Common Agricultural Policy (CAP) funds contribute to increasing the remuneration of family work (GFFI_CAP aid_) in pastoral systems (61.36 EUR goat^−1^), assimilating it to the rest of the systems. In the case of eco-efficiency indicators, significant differences are observed between groups in total market revenues in relation to non-renewable cumulative energy demand (EI1) because the level is clearly higher in pastoral systems (2.87 EUR GJ^−1^). Even if there are no significant differences between groups in GFFI with aid (EI2), the truth is that pastoral systems have higher levels of this indicator, while the lowest level is observed in intensive farms (IS) (0.31 EUR GJ^−1^).

## 4. Discussion

### 4.1. Energy Efficiency of Dairy Goat Farming from an Agroecological Perspective

Results show how, when analyzing outputs that have a market price, there are significant differences between production systems in relation to socialized energy (SO) in the form of milk and meat, mainly due to the higher milk productivity of intensive livestock farms (IS and ISC). However, from an agroecological approach, where other elements that are not traditionally evaluated, such as manure (reused on the farm), are included, the values of the outputs are equalized, with no significant differences between groups. Pérez-Neira et al. [47] estimated that when the energy from manure was considered, the energy output increased by 147%. The economistic bias in energy analyses distorts the understanding of energy metabolism by invisibilizing its main energy output and its relevance in fertilization [48]. Thus, manure in extensive systems constitutes a fundamental energy and biomass recirculation that contributes to the productive capacity and maintenance of the ecological functions of agroecosystems [50]. When analyzing the energy inputs coming from outside the farms, it is observed that there are also significant differences between the various production systems, particularly in relation to the use of concentrates and fodder for animal feed (also in Pollaro et al. [44] or Zucali et al. [45]), which is significantly higher in livestock farms that do not graze their animals. Adapting our functional unit to liters, our results show a slightly higher NR CED (IS, ISC, and GS), which is of the same order of magnitude (PS) as that obtained by Kanyarushoki et al. [59] for goat milk in France (ca. 10.5 and 7.0 vs. 7.9 MJ L^−1^). Applying an agroecological approach, it is possible to observe how internal energy flows, i.e., those occurring within the farm, can represent up to 50% of the CED in the case of pasture systems.

In other words, combining grazing with rational use of purchased feed reduces dependence on the use of non-renewable energy (NR CED) [49,51]. Thus, Soares-Cabral et al. [46] discuss the Brazilian case of how the partial replacement of concentrates, particularly soybean, with hay or grass can reduce dependence on external energy by 40%. The analysis of energy indicators under an agroecological approach shows a higher energy efficiency (final and NR EROI) in pastoral livestock systems (PS). In a context such as the current one, marked by climate change [28] and, particularly, by fossil fuel depletion [26,27], this greater efficiency has important repercussions. First, it decreases the direct GHG emissions in pastoral farms by reducing dependence on concentrates and oil and gas derivatives in all processes. This is especially true when carbon sequestration [38,40] and/or the introduction of organic livestock management practices [39] are considered. On the other hand, it cuts down the use of products that, in a geostrategic context such as the current one, may become more scarce or difficult to access, such as fertilizers or others. Finally, grazing and reuse of manure avoid energy costs associated with fertilization and animal feed (AECM or AECP) and with territorial costs (ALCP) while allowing the use of energy resources with territorial opportunity costs that can be more efficiently transformed into food (food/feed EROI) [47].

### 4.2. Economic Assessment of Dairy Goat Farming

The monetary data show that the PS group is penalized by the markets despite being the most energy-efficient, while intensive (IS and ISC) farms are rewarded in terms of value-added and family farm income. Although some research indicates that low-input livestock strategies may lead to better economic results and environmental improvements at the same time [13,22,58], the data of our study show that there is a contradiction between environmental sustainability and economic remuneration on market terms, in line with what Ripoll-Bosch et al. [60] found in their work on Mediterranean sheep farms.

The farms in the ISC group register the best monetary results in all indicators. They are the ones that obtain the highest total value added and value added per unit of work, as well as the highest remuneration of family labor, despite not being the most energy-efficient model. By having their own farmland, they substantially decrease the total costs by reducing purchased feed, maintaining high milk yields, and maximizing CAP subsidies compared with other more or less intensive production systems. The farms in the IS group require high fixed-capital investments, which translates into a lower net value added. This puts them at similar levels to the GS group and close to the PS group, although still above them.

The PS group, with low input strategies, had the lowest total costs and the highest eco-efficiency, as already shown in other studies [13,17,51]. However, they also show the lowest returns to market-oriented production (socialized output) and market income, which results in a lower gross value added (GVA) compared with more intensive systems. The low fixed-capital costs (depreciation) of pastoral farms, which reduce the differences in NVA with other systems, imply greater resilience since the long-term productive capacity of these farms does not depend so much on external non-renewable resources [51]. In the present study, this is also reflected in indicators ER1 and ER2.

The results show that all production systems receive public subsidies in similar amounts, except the IS group (with lower amounts). Although it is necessary to recognize the general unsustainability implied by dependence on public subsidies in livestock systems [61,62], it should also be considered that CAP aids incomes are already supporting sustainable and energy-efficient goat extensification strategies, as in the case of the PS group and, to a lesser extent, the GS groups.

The net value added per worker is positive in all systems, although the highest remunerations are obtained in the ISC group and, to a lesser extent, the IS groups. When public subsidies are considered, the differences in GFFI per goat among systems are reduced, leaving the PS group on par with the IS group and with no statistically significant differences with the GS group, although the higher results of the ISC group are maintained. When taking subsidies (aid income) into consideration in the calculation of family farm income per worker, we see that pastoral farms reach higher remuneration than the GS and IS groups, which register the lowest level, while the ISC group continues to register the highest results. The economic viability of all the family dairy goat systems analyzed is evident, and they additionally generate hired employment. Therefore, the data show that intensification does not imply an absolute monetary advantage in all cases and that extensification is remunerative for family dairy farming and, therefore, economically viable [58]. These results contrast with the claims of non-economic viability found in studies that calculate profitability (or remuneration of capital) through the deduction of opportunity costs such as unpaid family labor, land rent, and variable and fixed capital interest [18,19,21] but whose data show that the GFFI, if calculated, is positive. It is important to take into consideration that family farm systems pursue, first of all, the remuneration of family factors, mainly labor, as a way of living in a context of limited full-time employment alternatives [63], and that they additionally generate hired employment in rural areas often affected by depopulation.

### 4.3. Limitations and Future Research

Finally, we would like to highlight some of the limitations of this research that can constitute future lines of work: (1) the sample could be expanded to achieve statistical representativeness of the sector; (2) a systematic collection of data on a diverse sample of goat farms and over a period of several years, would provide a better understanding of the repercussions of crises and trends, on the energy and economic efficiency of the farms; (3) the system boundaries could also be expanded to include the remaining stages up to consumption (cradle to fork approach) [64]; (4) the energy impact of goat farming could be analyzed in a complementary way using other environmental categories related to, for example, water use, acidification, GHG emissions, etc. [46]; (5) the environmental functions associated with grazing could be monetarily valued to include them in cost-benefit analyses [65]; and (6) other socio-economic and cultural aspects (e.g., gender relations) could be included to assess the sustainability of livestock practices [66,67].

## 5. Conclusions

Energy indicators are increasingly relevant to assess dairy goat farming in the present context of climate change and the energy crisis. This study evidences the importance of internal energy flows as pasture and manure in the environmental performance of farms as they allow the calculation of costs and benefits in terms of avoided costs (AECM and AECP) of the different productive systems. These indicators are new in the comparative analysis of the sustainability of goat systems with different feeding managements and intensification levels. In addition, the economic results presented in this study conclude that the net gross value (NGV) and goat family farm income (GFFI), especially the NGV per worker and GFFI CAP aid per worker, are the accurate indicators to assess the economic viability of dairy goat family farms instead of profitability by contrast to the recent literature on this topic. Combining energy and monetary analysis, this study proposes two new indicators of energy efficiency (EI1 and EI2) that, in our results, prove the higher energy efficiency of dairy goat pastoral farming.

This work combines, in a novel way, the monetary and energetic aspects in the analysis of dairy goat production systems and contributes to broadening the knowledge of their multi-functionality. The results reveal that, from an economic and production-based point of view, stabling and the intensification of livestock systems facilitate food and reproduction management and contribute to increasing milk production and, consequently, income despite their higher production costs and capital investments. Although all systems studied can remunerate family labor and contribute to the generation of local employment, in market terms, the intensification strategy, together with an increase in food autonomy through the cultivation of their own crops for goat feed, results in more profitable and remunerative. Nevertheless, the analysis of energy indicators under an agroecological approach shows that pastoral farms are more energetically efficient.

The production-based perspective has proved to be valid in the context of affordable raw materials and other inputs, with a cost structure that does not include elements related to the use of natural capital. Currently, the agricultural sector faces a different reality, where factors related to the climate crisis or the energy transition are clearly affecting their functioning. Given that energy is also a key factor for explaining ecosystem processes, the analysis of energy indicators under an agroecological approach, that takes into consideration the internal energy flows, is indispensable and can contribute to give more active support to the pastoral systems, including the organic livestock, and avoiding the usual tendency to intensify management systems. The incorporation of these results in public aid payment schemes, such as eco-schemes, can contribute positively to the maintenance of pastoral goat systems, which are currently in the minority.

## Figures and Tables

**Table 1:** Main characteristics of the dairy goat production systems analyzed.

Production System	Main Characteristics
Indoor systems without associated crops (IS)	- Goats permanently indoors with outdoor exercise yards; - No grazing or crop surfaces associated with the goats; - Food supplied comes from outside the farm; - High consumption of concentrates and fodder; - Highly milk-productive, with de-seasonalized sales.
Indoor systems with associated crops (ISC)	- Goats permanently indoors with outdoor exercise yards; - Crop surfaces associated with the goats for fodder production; - High consumption of concentrates (from outside the farm) and fodder; - Highly milk-productive, with de-seasonalized sales.
Grazing systems with high feed supply (GS)	- Variable times of grazing; - Large surfaces dedicated to grazing, whether natural or cultivated; - High external supply of concentrates and even fodder; - Highly milk-productive, certain degree of seasonality.
Pastoral systems (PS)	- Herds outside the whole year, with variable grazing times; - Large surfaces dedicated to grazing, both natural and cultivated; - Feed from outside the farm is in short supply; - Lower milk production than in other groups marked seasonality; - Usually, multispecies livestock farms.

**Table 2:** Energy outputs of the four dairy goat production systems identified in Andalusia (GJ LSU^−1^). IS: indoor systems without associated crops; ISC: indoor systems with associated crops; GS: grazing systems with high feed supply; and PS: pastoral systems.

Energy Outputs	IS	ISC	GS	PS	*p*-Value
Total energy output (TEO) (a + b + c)	26.20	27.71	28.70	24.73	0.151
(a) Socialized output (SO) (i + ii)	9.70 ^a^	8.86 ^ab^	9.38 ^a^	5.60 ^b^	0.041
(i) Milk	9.52 ^a^	8.64 ^ab^	9.15 ^a^	5.34 ^b^	0.041
(ii) Meat	0.18	0.22	0.23	0.26	0.509
(b) Energy output reused (EOr) (i + ii)	16.41 ^b^	18.84 ^a^	19.35 ^a^	19.16 ^a^	0.030
(i) Inside the farm (ia + ib)	0.00 ^b^	1.66 ^b^	14.90 ^a^	18.96 ^a^	0.010
(ia) Milk	0.00 ^b^	0.00 ^ab^	0.50 ^ab^	0.70 ^a^	0.007
(ib) Manure	0.00 ^b^	1.66 ^b^	14.39 ^a^	18.27 ^a^	0.010
(ii) Outside the farm (manure)	16.41 ^a^	17.19 ^a^	4.45 ^b^	0.20 ^b^	0.006
(c) Increase/decrease in livestock (I/D L)	0.10	0.01	−0.04	−0.04	0.105

^a, b^ Values with different letters on the same row mean significant difference.

**Table 3:** Energy inputs of the four dairy goat production systems identified in Andalusia (GJ LSU^−1^). IS: indoor systems without associated crops; ISC: indoor systems with associated crops; GS: grazing systems with high feed supply; and PS: pastoral systems.

Energy Inputs	IS	ISC	GS	PS	*p*-Value
Cumulative energy demand (CED) (a + b)	111.45 ^a^	97.42 ^ab^	102.53 ^a^	74.27 ^b^	0.001
(a) External inputs (EI) (i + … + vi)	111.22 ^a^	74.25 ^b^	89.24 ^b^	36.90 ^c^	0.000
(i) Electricity	2.58	1.94	2.82	0.89	0.339
(ii) Petrol/gas	2.48	1.79	2.04	0.99	0.511
(iii) Feed purchased	99.01 ^a^	58.83 ^c^	78.78 ^b^	30.82 ^d^	0.000
(iv) Other operational expenses	3.60	7.30	3.43	3.40	0.282
(v) Labor	0.39	0.30	0.47	0.28	0.121
(vi) Machinery	3.16 ^ab^	4.09 ^a^	1.69 ^ab^	0.52 ^b^	0.006
(b) Internal inputs (II)	0.23 ^c^	23.17 ^b^	13.29 ^b^	37.37 ^a^	0.000
(vii) Cultivated forages	0.00 ^b^	23.17 ^a^	0.00 ^b^	1.36 ^b^	0.001
(viii) Cultivated grazing surfaces	0.23	0.00	4.90	6.46	0.076
(ix) Natural grazing surfaces	0.00 ^c^	0.00 ^c^	8.39 ^b^	29.55 ^a^	0.000
Non-renewable CED (i + … + vi)	31.87 ^a^	28.51 ^a^	28.27 ^a^	11.66 ^b^	0.007

^a, b, c, d^ Values with different letters on the same row mean significant difference.

**Table 4:** Energy efficiency indicators of the four dairy goat production systems identified in Andalusia. IS: indoor systems without associated crops; ISC: indoor systems with associated crops; GS: grazing systems with high feed supply; and PS: pastoral systems.

Energy Efficiency Indicators	Unit	IS	ISC	GS	PS	*p*-Value
Final EROI ^(1)^	-	0.24 ^c^	0.29 ^b^	0.28 ^b^	0.34 ^a^	0.001
NR Final EROI ^(2)^	-	0.83 ^b^	0.99 ^b^	1.04 ^b^	2.43 ^a^	0.002
NR EROI_food_	-	0.30 ^b^	0.31 ^ab^	0.34 ^ab^	0.54 ^a^	0.017
Dependence on external animal feed	%	99.66 ^a^	66.82 ^c^	82.55 ^b^	36.57 ^d^	0.000
AECM ^(3)^	GJ LSU^−1^	2.12 ^b^	2.10 ^b^	2.46 ^a^	2.62 ^a^	0.009
AECP ^(4)^	GJ LSU^−1^	0.00 ^b^	0.00 ^b^	13.02 ^b^	52.12 ^a^	0.000
	MJ L^−1^	0.00 ^b^	0.00 ^b^	1.68 ^b^	0.24 ^a^	0.000
Food/feed EROI	-	0.23	0.23	0.22	0.27	0.227
ALCP ^(5)^	ha LSU^−1^	0.00 ^b^	0.00 ^b^	0.74 ^b^	2.96 ^a^	0.000
	ha 1000 L^−1^	0.00 ^b^	0.00 ^b^	0.27 ^b^	1.64 ^a^	0.000

Where ^(1)^ final energy return on investment; ^(2)^ non-renewable final energy return on investment; ^(3)^ avoided energy cost of manure; ^(4)^ avoided energy cost of natural pasture consumed during grazing; and ^(5)^ avoided land cost of natural pasture consumed during grazing. ^a, b, c, d^ = values with different letters on the same row mean significant difference.

**Table 5:** Economic indicators of the four dairy goat production systems identified in Andalusia. IS: indoor systems without associated crops; ISC: indoor systems with associated crops; GS: grazing systems with high feed supply; and PS: pastoral systems.

Particulars	Unit	IS	ISC	GS	PS	*p*-Value
Economic						
SI ^(1)^	EUR goat^−1^	379.80 ^a^	327.03 ^a^	341.80 ^a^	196.48 ^b^	0.000
AI ^(2)^	EUR goat^−1^	9.31 ^b^	35.57 ^a^	24.60 ^a^	32.28 ^a^	0.030
IC ^(3)^	EUR goat^−1^	292.30 ^a^	226.35 ^b^	261.17 ^ab^	139.78 ^c^	0.000
HLC ^(4)^	EUR goat^−1^	18.04	25.60	28.32	25.34	0.878
AM ^(5)^	EUR goat^−1^	15.26	11.83	7.20	2.30	0.300
GVA ^(6)^	EUR goat^−1^	102.76	123.47	92.50	59.00	0.360
NVA ^(7)^	EUR goat^−1^	87.50	111.63	85.30	56.70	0.460
NVA _per worker_	EUR AWU^−1^	17,914	29,817	13,074	13,620	0.190
GFFI ^(8)^	EUR goat^−1^	54.24	74.22	49.59	29.08	0.560
GFFI_CAP aid_^(9)^	EUR goat^−1^	63.55	109.79	74.19	61.36	0.440
GFFI_CAP aid per worker_	EUR family AWU^−1^	20,533	47,135	23,463	28,104	0.143
Eco-efficiency						
EI1 ^(10)^	EUR GJ^−1^	1.82 ^b^	1.73 ^b^	1.83 ^b^	2.87 ^a^	0.016
EI2 ^(11)^	EUR GJ^−1^	0.31	0.56	0.38	0.98	0.060

Where ^(1)^ income from socialized outputs; ^(2)^ aid income; ^(3)^ intermediate consumption; ^(4)^ hired labor cost; ^(5)^ amortizations; ^(6)^ gross value added; ^(7)^ net value added; ^(8)^ goat family farm income; ^(9)^ goat family farm income with aid; ^(10)^ non-renewable cumulative energy demand in relation to total market revenues; and ^(11)^ non-renewable cumulative energy demand in relation to goat family income with aid. ^a, b, c^ Values with different letters on the same row mean significant difference.

## Data Availability

Data is contained within the article.

## Supplementary Materials

The following supporting information can be downloaded at https://www.mdpi.com/article/10.3390/ani14010104/s1, Figure S1: Main characteristics of the surfaces used for goats in systems reserving an area for grazing; Table S1: General characteristics, main outputs obtained, and inputs used during 2018 in the twenty-one farms monitored. IS: indoor systems without associated crops; ISC: indoor systems with associated crops; GS: grazing systems with high feed supply; and PS: pastoral systems.
