# Supplementary material for: Energetic and Monetary Analysis of Efficiency in Family-Owned Dairy Goat Production Systems in Andalusia (Southern Spain)
Source: Animals (Basel). 2023 Dec 27;14(1):104. doi: 10.3390/ani14010104 (PMC10778047; doi:10.3390/ani14010104)
Supplement: Supplementary file 1 [file animals-14-00104-s001.zip › animals-2746003-supplementary.pdf]

Supplementary material

Table S1. General characteristics, main outputs obtained and inputs used during 2018 in the twenty-one farms monitored. IS: Indoor systems without associated crops; ISC: Indoor systems with associated crops; GS: Grazing systems with high feed supply; PS: Pastoral Systems.

Item	Unit	IS	ISC	GS	PS	*p*
Number of goats	-	388^{b}	699^{a}	254^{b}	501^{ab}	*
LSU[(1)]	-	69.34^{b}	122.53^{a}	47.62^{b}	96.08^{ab}	*
Grazed surfaces[(2)]	ha goat $^{-1}$	0.00^{b}	0.00^{b}	0.29^{b}	0.74^{a}	***
Fodder cultivated surfaces[(3)]	ha goat $^{-1}$	0.00^{b}	0.07^{b}	0.00^{b}	0.00^{a}	**
Total work[(4)]	AWU	1.89	2.65	1.65	1.87	*ns*
Hied work[(4)]	AWU	0.58	1.00	0.57	0.68	*ns*
Family work[(4)]	AWU	1.31	1.65	1.08	1.18	*ns*
Work associated with activity	goats AWU $^{-1}$	207	272	164	280	*ns*
**(a) Outputs**						
Milk[(5)]	litres goat $^{-1}$	560.71^{a}	474.05^{a}	492.64^{a}	309.98^{b}	**
Meat[(6)]	kg goat $^{-1}$	5.63	4.32	5.41	5.82	*ns*
**(b) Inputs**						
Concentrates[(7)]	kg goat $^{-1}$	499.35^{a}	464.53 ^{a}	567.92^{a}	258.21^{b}	**
Fodder[(8)]	kg goat $^{-1}$	490.34^{a}	342.23^{ab}	191.43^{bc}	40.95^{c}	**
Electricity	kilowatt goat $^{-1}$	47.07	27.60	41.91	13.29	*ns*
Petrol	litres goat $^{-1}$	5.87	2.91	6.22	2.46	*ns*
Labor[(9)]	hours goat $^{-1}$	67.31	49.47	83.77	48.52	*ns*

[(1)] LSU=livestock standard unit, one adult equals 0.15 LSU; rest or categories equals 0.11 LSU; [(2)] Grazed surfaces: all the surfaces used for goats for grazing, including cultivated or natural areas; [(3)] Fodder cultivated areas: areas destined to fodder supply indoors; [(4)] AWU: Annual Work Unit. [(5)] Sold milk; [(6)] Sold goat kids, expressed in kg of live weight; [(7)] Purchased concentrates, there are not cultivated concentrates; [(8)] includes purchased and cultivated fodder; [(9)] Includes hired and family work. ^{a,b,c} Values with different letters on the same row are different (*$p < 0.05$; **$p < 0.01$; ***$p < 0.001$).

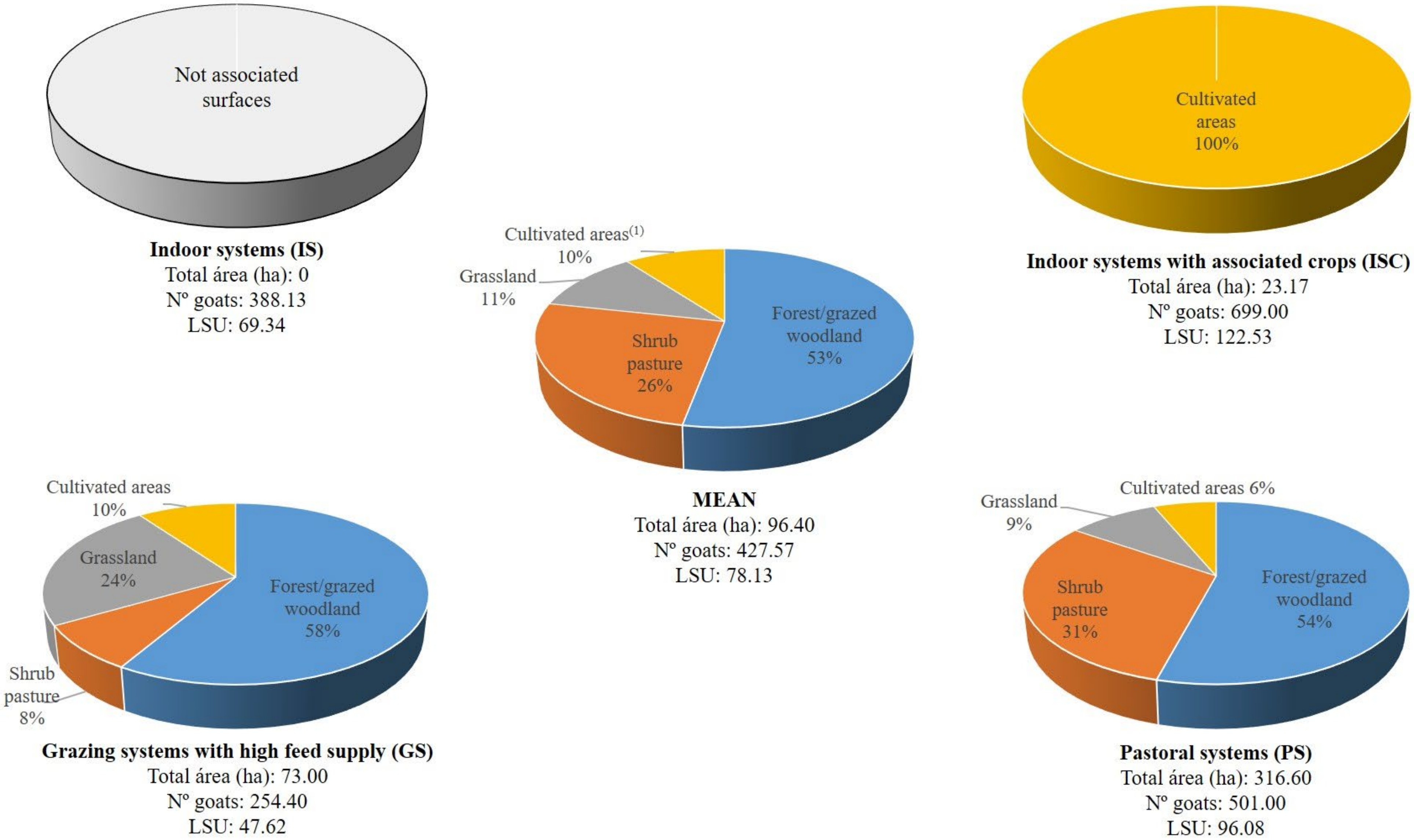

Figure S1. Main characteristics of the surfaces used for goats in the systems using surfaces for grazing. [(1)] Cultivated areas: includes surfaces for grazing and/or fodder
